# Apolipoprotein L1, income and early kidney damage

**DOI:** 10.1186/s12882-015-0008-6

**Published:** 2015-02-10

**Authors:** Ruth Tamrat, Carmen A Peralta, Salman M Tajuddin, Michele K Evans, Alan B Zonderman, Deidra C Crews

**Affiliations:** Department of Medicine, Johns Hopkins University School of Medicine, Baltimore, MD USA; Department of Medicine, University of California, San Francisco, CA USA; San Francisco VA Medical Center, San Francisco, CA USA; Laboratory of Epidemiology and Population Sciences, National Institute on Aging, National Institutes of Health, Baltimore, MD USA; Welch Center for Prevention, Epidemiology and Clinical Research, Johns Hopkins Medical Institutions, Baltimore, MD USA; Center on Aging and Health, Johns Hopkins Medical Institutions, Baltimore, MD USA; Division of Nephrology, Johns Hopkins University School of Medicine, Johns Hopkins Bayview Medical Center, 301 Mason F. Lord Drive, Suite 2500, Baltimore, MD 21224 USA

**Keywords:** *APOL1*, Chronic kidney disease, Socioeconomic status, African American, Renal, Albuminuria

## Abstract

**Background:**

The degree to which genetic or environmental factors are associated with early kidney damage among African Americans (AAs) is unknown.

**Methods:**

Among 462 AAs in the Healthy Aging in Neighborhoods of Diversity across the Life Span (HANDLS) study, we examined the cross-sectional association between apolipoprotein L1 (*APOL1*) risk variants and income with: 1) mildly reduced eGFR (<75 mL/min/1.73 m^2^, creatinine-cystatin C equation) and 2) elevated urine albumin-to-creatinine ratio (ACR) (≥17 in men and ≥25 mg/g in women). High risk *APOL1* status was defined by 2 copies of high-risk variants; low risk if 0 or 1 copy. Income groups were dichotomized as < $14,000/year (lowest income group) or ≥ $14,000/year. Logistic regression models were adjusted for age, sex, and % European ancestry.

**Results:**

Overall, participants’ mean age was 47 years and 16% (n = 73) had high risk *APOL1* status. Mean eGFR was 99 mL/min/1.73 m^2^. Mildly reduced eGFR was prevalent among 11% (n = 51). The lowest income group had higher adjusted odds (aOR) of mildly reduced eGFR than the higher income group (aOR 1.8, 95% CI 1.2-2.7). High-risk *APOL1* was not significantly associated with reduced eGFR (aOR 1.5, 95% CI 0.9-2.5). Among 301 participants with ACR data, 7% (n = 21) had elevated ACR. Compared to low-risk, persons with high-risk *APOL1* had higher odds of elevated ACR (aOR 3.8, 95% CI 2.0-7.3). Income was not significantly associated with elevated ACR (aOR 1.8, 95% CI 0.7-4.5). There were no significant interactions between *APOL1* and income.

**Conclusions:**

Both genetic and socioeconomic factors may be important determinants of early kidney damage among AAs.

**Electronic supplementary material:**

The online version of this article (doi:10.1186/s12882-015-0008-6) contains supplementary material, which is available to authorized users.

## Background

Apolipoprotein L1 (*APOL1*) belongs to a family of genes which encode for lipoproteins that are likely involved in the transport and metabolism of lipids. Unlike others in this gene family, *APOL1* possesses a signal sequence that allows for it to be exported out of the cell. Once extracellular, *APOL1* associates with HDL particles and may play a role in programmed cell death [1,2]. While they confer protection against the parasite Trypanosoma brucei, genetic variants in the *APOL1* region of chromosome 22 have been identified as major determinants of chronic kidney disease (CKD) in African Americans. The two most common high-risk variants of *APOL1*, G1 and G2, are only seen among individuals of West African descent, with approximately 12% of African Americans having two high-risk variants. In contrast, these high-risk variants are virtually nonexistent among Asians, Europeans, and Hispanics [3,4]. Multiple studies have now shown that, compared to those with zero or one high-risk variant, the presence of two high-risk variants is associated with advanced kidney disease in the setting of focal segmental glomerulosclerosis [3], hypertension-attributed nephropathy [5] and HIV-associated nephropathy [6]. High-risk *APOL1* is also associated with progression of established CKD, independent of diabetes status [7]. Less is known about the relation of *APOL1* and early kidney damage. Some studies have shown associations between having two 2 high-risk *APOL1* variants and prevalent high ACR [8] and very high ACR [9] among populations with largely preserved eGFR.

However, importantly, not all African Americans with two high-risk variants develop incident [10] or progressive CKD [7], suggesting that a “second hit” may be required. This second hit may be environmental, including infectious factors (as in the case of HIV associated nephropathy), as well as factors related to socioeconomic status (SES) or comorbid disease burden. Multiple studies have found that adjustment for SES attenuates observed disparities in kidney-related outcomes between African Americans and whites, suggesting that factors related to low SES contribute to the excess risk of advanced CKD and ESRD seen among African Americans [11,12]. Some studies have found that low SES correlates more strongly with CKD/ESRD among African Americans than whites, further supporting the notion of environmental contributions to racial disparities [13-15]. There are many possible mechanisms through which SES could contribute to these disparities, and several models have been proposed [11,16]. For example, limited availability of healthful foods in the neighborhoods of many low SES African Americans could lead to poor dietary patterns [17,18], which could ultimately affect CKD risk-- particularly given the growing body of evidence supporting that dietary patterns rich in fruits and vegetables could be kidney protective [19-21].

Despite recent advances in understanding genetic contributions to racial disparities in CKD, questions still remain, including the relation of genetic factors to the variation in GFR among populations largely free of established CKD [8]. Furthermore, the possible role of gene-environment interactions in determining early kidney disease among African Americans has not been well-explored. Therefore, the objective of this study was to evaluate whether genetic factors, environmental factors (SES), or their interaction are associated with markers of renal dysfunction among persons without established CKD in an urban population of predominantly low income African Americans.

## Methods

### Healthy Aging in Neighborhoods of Diversity across the Life Span (HANDLS): design and population

We examined cross-sectional data from the National Institute on Aging (NIA), HANDLS study. HANDLS is a community-based cohort study examining the influences of race and SES on the development of health disparities among minority and lower SES subpopulations. This cohort, recruited between 2004 and 2008, is comprised of self-identified African Americans and whites 30–64 years of age at baseline, from 13 Baltimore, MD neighborhoods selected to reflect socioeconomic and racial diversity. Each participant provided informed consent, and the National Institute of Environmental Health Sciences Institutional Review Board approved the study protocol [22].

The total HANDLS population is 3,720. For the purposes of our study, we restricted our analysis to African-American participants (N = 2200) who were genotyped for *APOL1* (N = 1024), underwent baseline serum cystatin C (N = 848) and serum creatinine measurements (N = 625), and reported their annual household income (final N = 462). Analyses of urinary albumin to creatinine ratio were restricted to participants who also underwent baseline urine assessments (N = 301).

### HANDLS: measurements and definitions

Demographic data, including age, sex, race, employment, educational and insurance status, and annual household income were obtained during an initial household survey by self-report. Poverty status was also determined at baseline and was based on reported annual household income below or above 125% of the 2004 US Department of Health and Human Services poverty guideline [23].

A health care provider in a mobile research vehicle obtained participants’ medical and social histories and performed physical examinations. Hypertension was defined as an average of seated and standing systolic blood pressure ≥140 mm Hg, an average of seated and standing diastolic blood pressure ≥90 mm Hg [24], a history of blood pressure medication use, and/or a self-report of a history of hypertension. Cardiovascular disease was defined as a self-report of a history of congestive heart failure, enlarged heart, angina, chest pain, myocardial infarction, coronary artery disease, mini-stroke, or cerebrovascular accident. Height and weight were measured and used to calculate BMI to determine the presence of obesity, defined as BMI ≥30.

Fasting venous blood specimens and spot urine samples were also collected in the mobile research vehicle and analyzed at the NIA Clinical Research Branch Core Laboratory (Baltimore, MD, USA) and Quest Diagnostics Inc. (Baltimore, MD and Chantilly, VA, USA). Diabetes mellitus was defined as a fasting plasma glucose concentration of ≥126 mg/dl (7.0 mmol/l), Hemoglobin A1c ≥ 6.5% or self-report of diabetes. Serum creatinine was measured at Quest Diagnostics Inc. by isotope dilution mass spectrometry (Olympus America Inc., Melville, NY, USA) and standardized to the reference laboratory at the Cleveland Clinic. Serum cystatin C assays were run in collaboration with the Department of Pathology at the University of Maryland on the Dimension Vista® system from Siemens (Glasgow, DE) using the Flex® reagent cartridge. The serum creatinine and cystatin C were used to estimate GFR using the combined creatinine-cystatin C equation (eGFR) [25,26]. Urinary albumin to creatinine measures were performed at Quest Diagnostics Inc. using an immmunoturbidimetric assay (Kamiya Biomedical Co., Seattle, Wash. USA).

Percent European ancestry was estimated with STRUCTURE software that uses genotype data to assign individuals to a population of origin. We entered 2000 ancestry informative markers into our model to estimate the percent European ancestry of study participants [27].

### *APOL1* Genotyping, imputation, and quality control

A total of 1024 study participants were successfully genotyped using the Illumina 1 M single-nucleotide polymorphism (SNP) genotyping array, passing inclusion criteria into the study. Initial inclusion criteria includes concordance between self-reported sex and sex estimated from X chromosome heterogeneity, > 95% genotype call rate per participant, concordance between self-reported African ancestry and ancestry confirmed by analyses of genotyped SNPs, and no cryptic relatedness to any other samples at a level of proportional sharing of genotypes > 15%. In addition, SNPs with Hardy-Weinberg equilibrium p-value > 1 x 10^−7^, missing by haplotype p-value > 1 x 10^−7^, minor allele frequency > 0.01, and call rate > 95% were included. Quality control was conducted using PLINK 1.06 [28]. Genotypes that passed quality control criteria were used for genotype imputation. Imputation was performed using MACH and minimac software (http://www.sph.umich.edu/csg/abecasis/mach/) based on phase 1 alpha freeze version 3 data of the 1000 Genomes Project multi-ethnic panel. Imputed SNP dosages and best guess genotypes for rs73885319, rs60910145, and rs71785313 in *APOL1* gene were extracted from the imputation dataset to generate G1 and G2 *APOL1* risk haplotypes. Imputation quality indicated by R^2^ estimates in MACH for rs73885319, rs60910145, and rs71785313 were 0.84, 0.84, and 0.94, respectively. An R^2^ > 0.30 indicates good accuracy of genotype imputation. The linkage disequilibrium (LD) values between rs73885319 and rs60910145 were r2 = 1.0 and D’ = 1.0, indicating the SNPs were in perfect LD.

### Exposures of interest

The two primary exposures we examined were *APOL1* and annual household income. Given previous studies have shown the association between risk of kidney disease and *APOL1* to follow a recessive mode of inheritance [3,6,7,9], we treated *APOL1* as a dichotomous variable with participants defined as low risk *APOL1* (0 or 1 high risk variant) or high risk *APOL1* (2 high risk variants).

We created quintiles of annual household income in order to better delineate differences in income, with 1 corresponding to the lowest quintile, because the majority of our cohort lived in poverty (72%). For the purposes of analysis, we divided these quintiles into two groups. The first group included those who fell into income quintiles <3, which corresponded to an annual household income < $14,000/year and from here on will be referred to as the ‘lowest income group’. The second group included those who fell into income quintiles ≥3, which corresponded to an annual household income ≥ $14,000/year, here on referred to as the ‘higher income group’.

### Outcome measures

The primary outcomes of our study were 1) mildly reduced eGFR, defined as eGFRcys < 75 mL/min/1.73 m^2^, as this threshold is associated with higher risk of ESRD, death and cardiovascular disease^2^ [25], and 2) high urinary albumin to creatinine ratio (ACR), defined as ACR ≥ 17 mg/g in men and ≥ 25 mg/g in women [29]. We also performed additional analyses of significantly reduced eGFR (<60 mL/min/1.73 m^2^) and high ACR (≥30 mg/g).

### Statistical analyses

We examined participant characteristics stratified by *APOL1* status and then by income status. To evaluate for any significant differences in these characteristics, t-tests were used for continuous variables, and Fisher’s exact tests were used for categorical variables.

We used multivariable logistic regression models to determine the relationship of *APOL1* and income with our outcomes. We created separate models for the association of *APOL1* with the outcome of interest and the association of income with the outcome of interest. Next, we created a combined model that included both *APOL1* and income as exposures, and then determined the association of each individual exposure with the outcome of interest. This combined model was then adjusted for age, sex, and % European ancestry. The unadjusted and adjusted models were tested for potential effect modification using interaction terms for *APOL1* × income. We also assessed the variance in early kidney damage explained by *APOL1* risk variants and income status by quantifying the difference in Nagelkerke’s R^2^ value from the adjusted logistic regression model (which included age, sex and % European ancestry) with and without the inclusion of *APOL1* or income.

### Sensitivity analyses

To assess whether our results varied by choice of primary measure of SES, we conducted several sensitivity analyses. We analyzed our primary outcomes (eGFR < 75 and albuminuria) substituting income, separately, with education level (dichotomized by high school diploma or equivalency), employment status (based on employment in the past month), health insurance coverage (yes versus no), and regular source of health care (yes versus no).

## Results

### Participant characteristics

A total of 462 HANDLS participants were included in our sample. Those that were not included were of similar age and sex as those included, however, poverty was much less prevalent among those not included (40%) as compared to those included (72%) in our analysis. Also, notably, a total of 16% of participants in our sample had high risk *APOL1* status, compared to 8% in the full sample (n = 1024) of African American HANDLS participants who underwent genotyping. The mean age of participants was 47.4 (SD 8.9) years and 57.4% were female (Table 1).

**Table 1:** **Participant characteristics by** ***APOL1*** **Risk group and income**

		***APOL1*** **Risk group**			**Income status**		
**Characteristic**	**n**	**Low-Risk (n = 389)**	**High-Risk (n = 73)**	**P value**	**Lowest Income <14,000/yr) (n = 185)**	**Higher Income (≥14,000/yr (n = 277)**	**P value**
**Demographics**							
Age (SD), years	462	47.6 (8.9)	45.9 (9.0)	0.123	47.9 (8.9)	47.0 (8.9)	0.253
Female, %	462	56.3	63.0	0.305	58.9	56.3	0.631
Percentage European Ancestry (SD), %	462	17.4 (8.5)	16.7 (8.7)	0.570	17.9 (9.3)	16.8 (8.0)	0.161
**Socioeconomic Factors**							
Less than high school education, %	462	33.9	30.1	0.590	30.8	35.0	0.366
Unemployed, %	411	40.9	39.3	0.888	66.3	23.3	<0.001
Uninsured, %	411	38.3	30.1	0.234	42.2	33.6	0.063
No regular health care source, %	411	37.7	36.1	0.886	45.8	31.8	0.005
**Clinical and Behavioral Factors**							
Hypertension, %	429	55.7	50.0	0.427	60.0	51.4	0.092
Systolic Blood Pressure, mean (SD), mmHg	450	123 (18)	121 (16)	0.534	122 (19)	122 (18)	0.891
Diabetes, %	462	24.9	19.2	0.370	20.5	26.4	0.182
Cardiovascular Disease, %	461	17.8	19.2	0.742	21.1	15.9	0.175
Total Serum Cholesterol, mean (SD), mg/dL	462	184 (48)	191 (43)	0.257	178 (42)	190 (51)	0.007
Obesity, %	457	41.3	38.9	0.794	34.4	45.3	0.025
Tobacco Use ≥ 100 cigs, %	435	70.5	69.6	0.886	76.8	66.3	0.023

When stratified by *APOL1* status, there were no significant differences in socio-demographic, clinical (hypertension, systolic blood pressure, diabetes, cardiovascular disease or total cholesterol) or behavioral factors. The median income in this sample fell between $17,500 and $20,000, and the majority of participants lived in poverty. When stratified by income above or below $14,000, those in the lowest income group were more likely to be unemployed and more likely to have smoked cigarettes than the higher income participants (P < 0.05 for both). Also, while non-significant, hypertension was more prevalent among the lowest income group. The higher income group had higher total cholesterol and obesity prevalence than the lowest income participants (P < 0.05 for both).

### Analyses of reduced eGFR

The mean eGFR of the sample was 99 mL/min/1.73 m^2^. Estimated GFR in the high-risk group was lower than in the low-risk *APOL1* group, but the difference was not statistically significant [96 (SD 23) versus 100 (SD 20); P = 0.2]. The overall prevalence of eGFR <75 mL/min/1.73 m^2^ was 11% (n = 51). Compared with persons with low-risk *APOL1*, high-risk persons were at 50% higher odds of eGFR < 75, but estimates did not reach statistical significance (Table 2). There was a significantly greater odds of reduced eGFR in the lowest income group compared to the higher income group that was consistent in unadjusted and adjusted models [adjusted odds ratio (aOR) 1.81, 95% Confidence Interval (CI) 1.21, 2.72; p =0.004]. There was no evidence of effect modification. Fully, 53% of participants with eGFR < 75 were among the lowest income group while only 22% were in the high risk *APOL1* group. The variance in eGFR < 75 explained by the inclusion of *APOL1* in our model adjusted for age, sex and % European ancestry was 0. 4% compared to 1.5% explained by the inclusion of income.

**Table 2:** **Odds of eGFR <75 or <60 ml/min/1.73 m** ^**2**^ **by** ***APOL1*** **risk group and income category, N = 462**

**Model – Variables**	**Odds Ratios for eGFR < 75 (95% Confidence Interval) n = 51 events**		**P interaction (** ***APOL1*** **x Income)**	**Odds Ratios for eGFR < 60 (95% Confidence Interval) n = 19 events**		**P interaction (** ***APOL1*** **x Income)**
	**High-risk versus Low-risk** ***APOL1*** **variants**	**Lowest Income versus Higher Income Groups**		**High-risk versus Low-risk** ***APOL1*** **variants**	**Lowest Income versus Higher Income Groups**	
**Individual Variables**						
APOL1	1.55 (0.91, 2.63)	-----		2.59 (2.48, 2.70)	-----	
Income	-----	1.80 (1.22, 2.67)		-----	1.09 (0.35, 3.41)	
***Additive Models***						
APOL1, Income Category	1.54 (0.91, 2.61)	1.80 (1.23, 2.63)	0.903	2.59 (2.47, 2.71)	1.08 (0.36, 3.22)	0.06
+ Age, Sex, Percent European ancestry	1.50 (0.90, 2.49)	1.81 (1.21, 2.72)	0.653	2.48 (2.29, 2.68)	1.06 (0.33, 3.36)	0.09

Estimated GFR <60 ml/min/1.73 m^2^ was prevalent among 4.1% of participants, and high-risk *APOL1* status was strongly associated with this outcome (aOR 2.48, 95% CI 2.29- 2.68; p = <0.001) while the lowest income group was not (aOR 1.06, 95% CI 0.31-3.65; p = 0.92), when compared to the low-risk *APOL1* and higher income groups, respectively. The variance in eGFR < 60 explained by the inclusion of *APOL1* in our adjusted model was 2.1% compared to 0.1% explained by the inclusion of income. There was some evidence of effect modification (p interaction 0.09 for APOL1 × income in our adjusted model) (Table 3). In unadjusted (due to the low event rate) models stratified by income group, high-risk *APOL1* was associated with greater odds of eGFR < 60 only in the lowest income group (OR 5.81, 95% 2.32-14.55). There was no statistically significant association with *APOL1* in the higher income group (OR 1.22, 95% 0.59-2.53).

**Table 3:** **Association of** ***APOL1*** **and income with sex-specific elevated albumin-to-creatinine ratio (ACR), N = 301**

**Model – Variables**	**Odds Ratios for Elevated ACR (95% Confidence Interval) n = 21 events**		**P interaction (** ***APOL1*** **x Income)**
	**High-risk versus Low-risk** ***APOL1*** **variants**	**Lowest Income versus Higher Income Groups**	
***Individual Variables***			
*APOL1*	3.69 (2.01, 6.78)	------	
Income	------	1.91 (0.72, 5.08)	
***Additive Models***			
*APOL1*, Income Category	3.56 (2.06, 6.16)	1.79 (0.68, 4.74)	0.811
+ Age, Sex, Percent European ancestry	3.76 (1.95, 7.25)	1.78 (0.70, 4.49)	0.727

### Analyses of elevated urinary albumin-to-creatinine

Among the 301 participants who provided a urine sample, the median urinary ACR was 0.8 mg/g [interquartile range (IQR) 0.50-2.60]. A total of 7.0% of participants had elevated ACR (defined by sex-specific cut points). High-risk *APOL1* status was strongly associated with greater odds of elevated ACR (aOR 3.76, 95% CI 1.95, 7.25); and there was a non-significant association of lower income and elevated ACR, with no evidence of effect modification (Table 4).

**Table 4:** **Sensitivity analysis**

**Model – Variables**	**Odds Ratios for eGFR < 75(95% Confidence Interval)n = 51 events**		**P interaction (** ***APOL1*** **x SES measure)**	**Odds Ratios for Elevated ACR(95% Confidence Interval) n = 21 events**		**P interaction (** ***APOL1*** **x SES measure)**
	**High-risk versus Low-risk** ***APOL1*** **variants**	**Low SES versus Higher SES Group**		**High-risk versus Low-risk** ***APOL1*** **variants**	**Low SES versus Higher SES Group**	
***Additive Models***						
*APOL1*, Education	1.60 (1.00, 2.57)	1.95 (1.50, 2.54)	0.4	3.89 (2.28, 6.63)	1.68 (0.34, 8.23)	0.823
*APOL1*, Employment	2.06 (1.53, 2.78)	4.64 (3.40, 6.33)	0.017*	3.12 (1.73, 5.63)	1.32 (0.83, 2.08)	0.894
*APOL1*, Health Insurance	1.92 (1.27, 2.90)	1.00 (0.53, 1.89)	0.662	3.04 (1.65, 5.60)	0.56 (0.14, 2.28)	**could not be determined
*APOL1*, Regular Health Care Source	1.91 (1.15, 3.17)	0.54 (0.31, 0.94)	0.871	3.09 (1.56, 6.13)	0.64 (0.14, 2.86)	**could not be determined

Note: Data on employment, health insurance and regular source of health care were missing for 11% in the eGFR < 75 models and for 17% of the ACR models. For the eGFR < 75 models, these variables were missing in 16% of the high-risk APOL1 group compared to 10% of the low-risk group. For the ACR models, these variables were missing in 25% of the high-risk APOL1 group compared to 15% of the low-risk group.

*High-risk *APOL1* was associated with eGFR < 75 only among those who were unemployed [3.54 (1.98, 6.35)] compared to the employed [0.45 (0.13, 1.58)].

**Imbalance in strata.

Association of *APOL1* and Other Indicators of Socioeconomic Status (SES) with eGFR < 75 ml/min/1.73 m^2^ or Sex-Specific Elevated Albumin-to-Creatinine Ratio (ACR).

Among persons with elevated ACR, 29% were in the high-risk *APOL1* group and 33% were in the lowest income group); and the majority had hypertension (85%) and/or diabetes (57%). The variance in elevated ACR explained by the inclusion of *APOL1* in our adjusted model was 5.7% compared to 1.5% explained by the inclusion of income. Subsequent inclusion of hypertension and diabetes to our primary model, separately, explained 8.9% and 7.5%, respectively, of the variance in elevated ACR.

A total of 11 (3.7%) participants had high ACR (≥30 mg/g). In unadjusted models (due to a low event rate), high-risk *APOL1* status was associated with high ACR, however estimates were not statistically significant (OR 3.19, 95% CI 0.55-18.59). Income was not associated with high ACR (OR 0.90, 95% CI 0.48-1.72).

### Sensitivity analyses

Our analyses substituting income for other measures of SES yielded results which were qualitatively similar to our primary analyses, except in the case of our inclusion of health insurance and a regular source of health care (Table 4). For these metrics of access to care, only high-risk *APOL1* was associated with greater odds of mildly reduced eGFR or elevated ACR. There was some evidence of effect modification noted, with high-risk *APOL1* only being associated with greater odds of eGFR < 75 among unemployed participants (p interaction = 0.017 for *APOL1* × employment status).

## Discussion

In this study, we evaluated a cohort of young African Americans largely free of established CKD to examine the relation of *APOL1*, income and early kidney disease. We found that income, but not high-risk *APOL1*, was significantly associated with mildly reduced kidney function. Upon examining further reductions in kidney function (eGFR < 60), the association of high-risk *APOL1* and reduced kidney function was significant. We also found that high-risk *APOL1* status, but not income, was significantly associated with elevated ACR. The associations of *APOL1* with markers of early kidney disease did not vary by income category, or vice versa.

Few other studies have examined the association of *APOL1* with milder forms of kidney disease and findings have varied. Foster, *et al.* [10] performed a prospective study examining a cohort of African Americans with no CKD at baseline and found an increased risk for eGFR < 60 and progression to ESRD among those with the high-risk genotype. Friedman, *et al.* [8] found a 3-fold increased risk of eGFR < 60 and microalbuminuria associated with high risk *APOL1* among nondiabetic African Americans in a cross-sectional study; however, this association did not persist among persons with diabetes. Freedman, *et al.* [9] examined these cross-sectional relationships in relatives of patients with non-diabetic ESRD. They found a significant association between *APOL1* and macroalbuminuria, but not with microalbuminuria or eGFR < 60. In aggregate, these studies and ours suggest that high-risk *APOL1* variants may be more strongly associated with advanced and progressive kidney disease than with early disease, arguing for a primary role of *APOL1* as a progression rather than initiation factor in CKD.

Recent advances have swung the pendulum of research and clinical thought regarding the etiology of racial disparities in kidney disease towards the role of genetic contributions. The results of our study suggest that genetic factors may not fully explain these disparities. In this sample largely free of CKD, a large proportion of individuals with early kidney damage were among those without high-risk *APOL1* variants. Rather, diabetes and/or hypertension were prevalent among the majority of participants with early kidney damage. Thus, despite the highly significant association between high-risk *APOL1* and kidney disease among African Americans in multiple recent studies [3,4,7,10], it may not be the only contributor to early kidney disease in this population. Other factor(s), including those related to and influenced by SES, may be the primary initiators of CKD among African Americans. In our study, for example, we found that only about a third of individuals with elevated ACR had high-risk *APOL1* variants, however, the overwhelming majority had hypertension. Therefore, early CKD identification and treatment of modifiable risk factors, such as hypertension, may offer the greatest opportunity for reducing CKD disparities among minority and low income populations.

Our finding of greater odds of eGFR <75 among the lowest compared to the higher income group is consistent with prior studies implicating SES as a major predictor of early kidney disease among African Americans [12,13]. Our analyses of other indicators of SES were generally consistent with the income analyses, except our finding of no association between access to care measures and eGFR < 75. Here, reverse causality was possible, as individuals in poor health (ie. with reduced kidney function and other chronic illnesses) may be more able to obtain public health insurance and may seek care more often than healthier persons. There are multiple possible mechanisms through which low SES could lead to increased risk of kidney disease, most of which have yet to be thoroughly investigated. Aside from its potential impact on receipt of high quality health care, there are less obvious mechanisms, related to the social environment and psychosocial impact of low SES that may contribute as well [30,31]. Future studies are warranted elucidating pathways between low SES and kidney disease.

The limitations of our study should be considered. First, its cross-sectional design disallows direct causal inferences regarding the associations examined, and thus, longitudinal studies of this kind are needed. Second, despite our stratifying participants around an annual income of $14,000, our study population was largely comprised of low income individuals, which may have influenced our results. Third, we lacked complete data on some variables included in our sensitivity analyses. Finally, our relatively low event rate for early kidney damage did not allow adjustment for multiple covariates, or more granular analyses of income levels; and unmeasured confounders, particularly of the association of income and early kidney disease, are likely. Our power to detect statistically significant differences was limited in some of our models (Additional file 1). We included adjustment for a parsimonious set of variables despite the low event rate, and found that this did not substantially alter the point estimates of our univariate models. While our findings can serve to generate hypotheses, larger studies more representative of all African Americans are warranted addressing these relationships among well-characterized populations with a greater range of kidney disease.

## Conclusions

Our study is one of few examining the association of both genetic and environmental factors with early kidney damage [32]. In a population of urban-dwelling, primarily low-income African Americans, we found that genetic factors were significantly associated with albuminuria and significantly reduced eGFR, however, income level was a correlate of less severe markers of early kidney damage. Thorough examination of the burden of CKD among African Americans may require consideration of both genetic as well as environmental factors.

## Additional file

Additional file 1:
**Power calculations for unadjusted models.**
