# Supplementary material for: Apolipoprotein L1, income and early kidney damage
Source: BMC Nephrol. 2015 Feb 10;16:14. doi: 10.1186/s12882-015-0008-6 (PMC4361142; doi:10.1186/s12882-015-0008-6)
Supplement: Additional file 1: — Power calculations for unadjusted models. [file 12882_2015_8_MOESM1_ESM.docx]

**Power Calculations for Unadjusted Models**

| **Outcome** | **Primary Exposure** | **Power to Detect Statistically Significant Differences at p<0.05** |
| --- | --- | --- |
| Estimated GFR <75 ml/min/1.73 m^2^ | High-risk versus Low-risk *APOL1* variants | 20% |
|  | Lowest Income versus Higher Income Groups | *45% |
| Estimated GFR <60 ml/min/1.73 m^2^ | High-risk versus Low-risk *APOL1* variants | *40% |
|  | Lowest Income versus Higher Income Groups | 3% |
| Sex-Specific Albuminuria | High-risk versus Low-risk *APOL1* variants | *70% |
|  | Lowest Income versus Higher Income Groups | 19% |

*Statistically significant results noted.
